# Advances in the Association Between the *ADRB2* Gene and Bronchial Asthma: Genetic Polymorphisms, Epigenetic Regulation, and Clinical Applications

**DOI:** 10.3390/cimb48050457

**Published:** 2026-04-29

**Authors:** Xiaolong Sun, Yang Li, Yiyao Bao

**Affiliations:** Department of Surgical Intensive Care Unit, Children’s Hospital, Zhejiang University School of Medicine, National Clinical Research Center for Children and Adolescents’ Health and Diseases, Hangzhou 310052, China; 6520070@zju.edu.cn (X.S.); 6523217@zju.edu.cn (Y.L.)

**Keywords:** *ADRB2* gene, bronchial asthma, genetic polymorphism, epigenetics, drug response, individualized therapy

## Abstract

Bronchial asthma is a common chronic respiratory disease with a complex etiology, wherein the interaction between genetic and environmental factors plays a critical role in its pathogenesis. The β-2 adrenergic receptor gene (*ADRB2*) is pivotal in regulating airway smooth muscle relaxation. Its genetic polymorphisms have been extensively studied and are closely associated with asthma susceptibility, clinical phenotypes, and drug responses. Recently, the relationship between major single-nucleotide polymorphisms (SNPs) of the *ADRB2* gene and bronchodilator efficacy, alongside its transcriptional regulation and epigenetic modification mechanisms, has been progressively elucidated. Furthermore, *ADRB2* genotype-guided therapeutic strategies have shown potential clinical and economic value in selected studies, but they remain exploratory and have not yet been incorporated into routine guideline-based asthma management algorithms. This review summarizes recent advances in the role of the *ADRB2* gene concerning genetic susceptibility, disease phenotyping, environmental interactions, and immune regulation in asthma, aiming to provide theoretical support and clinical guidance for precision diagnosis and treatment.

## 1. Introduction

Bronchial asthma represents a prevalent and highly heterogeneous chronic respiratory disorder, characterized by recurrent airway inflammation, bronchial hyperresponsiveness, and reversible airflow obstruction [1,2]. Although the absolute number of individuals affected by asthma has continued to increase with population growth and ageing, recent Global Burden of Disease 2021 analyses indicate that the global age-standardized incidence and prevalence of asthma have declined over the past three decades; nevertheless, asthma remains a major public health priority because of its substantial morbidity, recurrent exacerbations, and uneven burden across regions and age groups. The pathophysiology of asthma is exceedingly complex, primarily driven by intricate interactions between genetic predispositions and environmental stimuli.

The pathophysiology of asthma is exceedingly complex, primarily driven by intricate interactions between genetic predispositions and environmental stimuli. Amidst this complexity, emerging evidence highlights the pivotal role of genetic factors in dictating both disease susceptibility and inter-individual variability in pharmacological responses.

At the forefront of asthma pharmacogenomics is the β-2 adrenergic receptor gene (*ADRB2*), mapped to the human chromosome 5q31-q32 locus. This gene encodes the β-2 adrenergic receptor, a G-protein-coupled receptor that acts as a central mediator in airway smooth muscle relaxation [3,4]. Upon activation by endogenous catecholamines or exogenous agonists, the receptor initiates the intracellular cyclic adenosine monophosphate (cAMP) signaling cascade, which subsequently promotes profound bronchodilation and alleviates airway constriction. Consequently, β-2 agonists remain the cornerstone for the acute management of asthma exacerbations [5]. However, the therapeutic efficacy of these bronchodilators is heavily contingent upon the structural integrity, expression levels, and functional state of the *ADRB2* gene [6].

The *ADRB2* gene is highly polymorphic, with the +46A>G (Arg16Gly, rs1042713) and +79C>G (Gln27Glu, rs1042714) single-nucleotide polymorphisms(SNPs) being the most extensively investigated variants. These functional polymorphisms have been shown to significantly alter the receptor’s conformation, down-regulation dynamics, and coupling efficiency, thereby modulating patients’ clinical responses to standard β-2 agonists such as albuterol. Consequently, individuals harboring distinct allelic variants exhibit substantial discrepancies in receptor activity, drug tolerance, and overall therapeutic outcomes. The advent of molecular techniques, notably allele-specific polymerase chain reaction (AS-PCR), has facilitated the precise identification of these genotypes, laying the molecular groundwork for customized therapeutic interventions [7,8].

Driven by the profound correlation between *ADRB2* polymorphisms and variable drug responses, the paradigm of asthma management is increasingly shifting toward genotype-guided individualized therapy. Leveraging patient-specific genetic profiles to optimize drug selection and titrate dosages holds immense promise for maximizing clinical efficacy, mitigating adverse drug reactions, and alleviating the global economic burden of asthma care. Beyond simple genetic variation, recent advances have illuminated the roles of epigenetic modifications, such as DNA methylation and microRNA regulation, in further shaping *ADRB2* expression and asthmatic phenotypes. Therefore, an in-depth synthesis of the *ADRB2* gene’s biological functions, its genetic and epigenetic landscape, and its clinical implications is indispensable [9]. This comprehensive review aims to critically evaluate the current progress regarding the role of the *ADRB2* gene in asthma susceptibility, phenotypic variation, and pharmacogenomics, ultimately providing a robust theoretical framework and actionable insights to propel the clinical translation of precision medicine in asthma management [10].

## 2. Biological and Structural Foundations of *ADRB2*

### 2.1. Gene Localization, Protein Structure, and Conformational Dynamics

The β-2 adrenergic receptor (β2AR), encoded by the *ADRB2* gene, is a prominent member of the G protein-coupled receptor (GPCR) superfamily. It is extensively involved in diverse physiological processes, most notably mediating airway smooth muscle relaxation within the respiratory system. The human *ADRB2* gene is mapped to the chromosome 5q31-q32 locus [10]. This specific genomic region harbors a cluster of genes associated with immune and inflammatory responses, thereby conferring a critical genetic basis for susceptibility to respiratory disorders such as bronchial asthma [10].

Structurally, the β2AR protein features a canonical architecture comprising seven transmembrane alpha-helices (TM1 to TM7), which form the hydrophobic core of the receptor. The N-terminus is positioned extracellularly, while the C-terminus resides within the intracellular space. The intracellular C-terminal tail serves as a vital regulatory domain for signal transduction and is subject to various post-translational modifications, including phosphorylation and ubiquitination [11]. These modifications intricately govern receptor desensitization, internalization, and downstream signaling cascades.

The highly specialized structural topology of β2AR enables it to detect the binding of adrenergic ligands and subsequently undergo dynamic conformational transitions to activate intracellular signaling pathways. Structural biology studies have elucidated that transmembrane helix 6 (TM6) undergoes a substantial outward displacement, approximately 6 to 14 Angstroms, during receptor activation [12]. This prominent conformational shift is an absolute prerequisite for the efficient coupling and subsequent activation of the stimulatory G protein (Gs). Furthermore, the extracellular loops of the receptor participate actively in signal regulation. Specifically, phenylalanine 193 (F193) located within the second extracellular loop (ECL2) plays a pivotal role in coordinating the binding of β-arrestin [13]. Mutagenesis studies indicate that alterations at this residue selectively attenuate β-arrestin recruitment, thereby modulating signaling bias and fine-tuning intracellular cascades [13]. This highlights that the extracellular domains are not solely responsible for ligand recognition but also integral to the precise orchestration of downstream signaling.

Molecular docking and molecular dynamics simulations have further characterized the diverse binding paradigms and energetic profiles of β2AR interacting with various ligands. Typical endogenous agonists, such as epinephrine and norepinephrine, as well as exogenous bronchodilators like albuterol, anchor into the orthosteric binding pocket by forming a robust hydrogen bond network. Key amino acid residues involved in these interactions include aspartate 113 (D113), asparagine 312 (N312), and serine residues S203, S204, and S207 [14]. This intricate interaction network ensures high-affinity ligand binding and robust receptor activation [14]. Additionally, β2AR exhibits basal, spontaneous activity, meaning a small proportion of the receptors exist in an active conformation even in the absence of a ligand [12]. This basal activity provides the structural rationale for the efficacy of inverse agonists, which function by stabilizing the inactive conformation and suppressing this spontaneous activation [12].

To summarize the intricate relationship between the structural domains of β2AR and their physiological roles, the key functional regions and critical amino acid residues are detailed in Table 1.

### 2.2. Signal Transduction and Transcriptional Regulation

**Figure 1:**
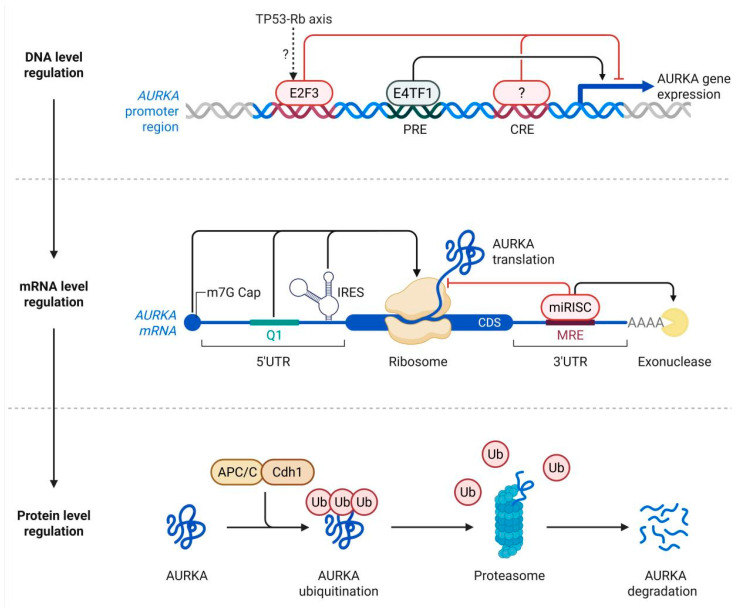
The Molecular Structure, Signal Transduction, and Transcriptional Regulation of *ADRB2*, including E2F1-mediated transcriptional activation. E2F1 is shown as the transcription factor directly regulating *ADRB2* promoter activity based on the studies discussed in the text.

As a quintessential member of the GPCR family, the β2AR orchestrates complex intracellular signaling cascades primarily through its interaction with heterotrimeric G proteins. The canonical signal transduction mechanism of β2AR predominantly relies on its coupling with the stimulatory Gs. Upon ligand-induced activation, the Gs alpha subunit dissociates and stimulates the membrane-bound effector enzyme adenylyl cyclase. This enzyme subsequently catalyzes the conversion of adenosine triphosphate into cAMP, drastically elevating its concentration within the intracellular milieu. Acting as a critical second messenger, cAMP binds to and activates protein kinase A (PKA). The activated PKA subsequently phosphorylates a myriad of downstream target proteins. In the specific context of airway smooth muscle cells, PKA-mediated phosphorylation effectively inhibits the activity of myosin light chain kinase (MLCK), which inherently diminishes intracellular calcium sensitivity and directly precipitates the profound relaxation and dilation of the airways [10,15], as shown in Figure 1.

Beyond the classical Gs-cAMP-PKA axis, the β2AR signaling network demonstrates remarkable pleiotropy and spatial-temporal plasticity, significantly regulated by β-arrestin-mediated biased signaling. Following initial activation, the receptor is rapidly phosphorylated by G protein-coupled receptor kinases, creating high-affinity binding sites for β-arrestins. While β-arrestins canonically mediate receptor desensitization and clathrin-dependent internalization, they also function as versatile scaffolding proteins that initiate parallel, G protein-independent signaling cascades. Most notably, β-arrestin recruits components of the mitogen-activated protein kinase (MAPK) pathway, thereby transmuting extracellular stimuli into transcriptional responses that govern cellular proliferation, differentiation, and apoptosis [13,16]. The inherent dynamism of this signal transduction is further underscored by single-molecule fluorescence spectroscopy, which reveals that transmembrane helix VII (TM VII) of the β2AR spontaneously fluctuates between inactive and active-like conformations even in the absence of ligands. The binding of specific ligands profoundly modulates the kinetic parameters of this conformational exchange, ultimately dictating both the intensity and the bias of the downstream signal [16]. Furthermore, the diverse post-translational modifications of the receptor’s intracellular C-terminal tail, including phosphorylation and ubiquitination, govern its interaction with β-arrestin, precisely calibrating the duration of the signal and the trajectory of endocytic trafficking [11].

In pathological states such as bronchial asthma, the fidelity of these signaling pathways is frequently compromised. Genetic polymorphisms within the *ADRB2* gene, notably Arg16Gly and Gln27Glu, profoundly alter receptor coupling efficiency and subsequent drug responsiveness, directly culminating in the clinically observed variability in the bronchodilator efficacy of β2-agonists like albuterol [10,17]. Additionally, the overall signal output is inextricably linked to the absolute abundance of the receptor, which is subject to rigorous epigenetic modifications, particularly DNA methylation, operating as a fundamental determinant of baseline receptor expression [18].

While post-translational modifications and conformational dynamics dictate the immediate signaling kinetics of β2AR, the basal and inducible expression of the receptor is meticulously controlled at the transcriptional level. Recent advances have identified the transcription factor E2F1, classically known for its pivotal role in cell cycle progression, as a critical positive regulator of *ADRB2* gene expression. Experimental investigations utilizing cloned human *ADRB2* 5′ regulatory regions in lung adenocarcinoma (A549) and human bronchial epithelial (BEAS-2B) cell lines have definitively elucidated this direct regulatory mechanism. The exogenous overexpression of E2F1 significantly amplifies the promoter-driven reporter activity of the *ADRB2* gene, whereas the targeted knockdown of E2F1 results in a precipitous decline in promoter function. Furthermore, site-directed mutagenesis of the putative E2F1 binding sites within the promoter robustly attenuates this transcriptional enhancement, verifying the high specificity of the interaction. Chromatin immunoprecipitation (ChIP) assays have provided definitive in vivo confirmation of the physical binding between E2F1 and the *ADRB2* promoter architecture [19]. Accordingly, E2F1 rather than E2F3 or E4TF1 should be represented in schematic illustrations of *ADRB2* transcriptional regulation.

The physiological implications of E2F1-mediated transcriptional regulation are profound. Quantitative real-time PCR (RT-qPCR) and Western blot analyses have corroborated that the targeted modulation of E2F1 corresponds directly to proportional alterations in both *ADRB2* messenger RNA transcripts and functional β2AR protein levels. Given the indispensable role of the β2AR in mitigating airway hyperreactivity and modulating local inflammatory responses, the dynamic regulation of its expression by E2F1 constitutes a critical nexus in the pathogenesis of respiratory disorders. E2F1-driven transcriptional tuning may serve as a fundamental mechanism underlying asthma susceptibility and exacerbation severity, thereby presenting a highly promising upstream target for novel, gene-directed therapeutic interventions [19].

To synthesize the multifaceted molecular mechanisms governing the β2AR, the primary signaling pathways and transcriptional regulators are summarized in Table 2.

### 2.3. Expression Profile in the Respiratory and Immune Systems

The *ADRB2* exhibits a ubiquitous yet highly specialized expression profile throughout the respiratory system, most prominently localized within the architectural framework of bronchial smooth muscle cells and a diverse array of resident and circulating immune cell populations. Within the bronchial smooth muscle, *ADRB2* operates as the primary molecular interface for endogenous catecholamines, meticulously orchestrating the dynamic equilibrium between muscular relaxation and contraction. Consequently, it serves as the principal pharmacological target for clinically administered bronchodilators in the management of obstructive airway diseases [20]. Furthermore, spatial-temporal expression analyses reveal that *ADRB2*, alongside critical downstream signaling effectors such as adenylyl cyclase (AC) and phosphodiesterase (PDE), is robustly expressed in pulmonary tissues during neonatal and early developmental stages. This sustained transcriptional upregulation strongly suggests that *ADRB2* is indispensable for normal lung morphogenesis, functional maturation, and the lifelong maintenance of pulmonary physiological integrity [21].

Beyond its foundational role in airway mechanics, the widespread distribution of *ADRB2* across various immune cell lineages underscores its integral function in immunological modulation. Significant expression levels of *ADRB2* have been identified in natural killer (NK) cells, monocytes, and peripheral blood mononuclear cells (PBMCs). The heterogeneous expression density observed across these distinct immune subsets implies a highly specialized role in calibrating cell-specific immune responses and maintaining immune homeostasis [22]. Specifically, in the context of allergic diatheses such as bronchial asthma, the expression profile of *ADRB2* within pulmonary immune reservoirs undergoes pronounced dynamic alterations. For instance, *ADRB2* expression is markedly upregulated in antigen-presenting cells, particularly dendritic cells (DCs), a phenomenon that is inextricably linked to the underlying pathogenesis of allergic airway inflammation [23]. Additionally, *ADRB2* is abundantly expressed on the surface of alveolar macrophages, where it exerts profound regulatory effects on local immune responses by modulating the cAMP-PKA signaling cascade, thereby actively participating in the resolution or exacerbation of pulmonary infections and local inflammatory processes [24].

The duality of *ADRB2* expression in both structural airway cells and diverse immune cell populations positions it as a central molecular determinant in the pathogenesis of bronchial asthma, directly influencing both airway hyperresponsiveness and chronic inflammation. The *ADRB2*-mediated dilation of bronchial smooth muscle constitutes the primary physiological mechanism for the acute relief of asthma symptoms, firmly establishing β-2 agonists as first-line therapeutic agents. However, the efficacy of this mechanism is heavily modulated by *ADRB2* genetic polymorphisms (e.g., Arg16Gly and Gln27Glu), which significantly dictate individual disease susceptibility and pharmacotherapeutic responsiveness. Specific allelic variants of these loci have been clinically demonstrated to predispose patients to refractory or difficult-to-control asthma phenotypes, highlighting the genetic complexity underlying structural airway responses [25].

In parallel with its mechanical functions, the immunomodulatory capacity of *ADRB2* constitutes a secondary, equally critical axis in asthma pathogenesis. Experimental asthma models have demonstrated that the localized upregulation of *ADRB2* within pulmonary DCs and group 2 innate lymphoid cells (ILC2s) vigorously promotes the activation of these innate immune effectors. This aberrant activation accelerates the localized release of potent inflammatory mediators, thereby exacerbating the overarching state of airway inflammation and tissue remodeling [23,26]. Conversely, targeted pharmacological blockade of *ADRB2* has been shown to effectively attenuate the pulmonary infiltration of inflammatory cells and progressively diminish the localized populations of T helper 2 (Th2) cells and regulatory T cells (Tregs), ultimately conferring substantial symptomatic relief and reducing disease severity in asthmatic models [23].

Recent neuroimmunological investigations have additionally highlighted the involvement of *ADRB2* in a complex neuroimmune reflex arc. Specifically, within the context of the brain-lung axis, norepinephrine released by the sympathetic nervous system directly activates *ADRB2* receptors situated on pulmonary interstitial macrophages. This specific neuro-immune interaction profoundly amplifies the local inflammatory cascade and can precipitate severe pulmonary tissue injury, revealing a novel mechanism by which psychological or physiological stress may worsen asthma outcomes [27]. Collectively, these findings underscore that the dual functionality of *ADRB2* across bronchial smooth muscle and localized immune cell populations profoundly governs both the onset and the progressive deterioration of asthma, thereby reinforcing its status as an indispensable and multifaceted therapeutic target.

## 3. Genetic and Epigenetic Determinants of Asthma Susceptibility

### 3.1. Major Functional Polymorphisms (Arg16Gly and Gln27Glu)

The *ADRB2* gene is characterized by extensive genetic heterogeneity, with the two most clinically significant and rigorously investigated functional single-nucleotide polymorphisms (SNPs) being Arg16Gly (rs1042713, +46A>G) and Gln27Glu (rs1042714, +79C>G). The distribution of these allelic variants exhibits profound ethnic and geographic stratification. Epidemiological investigations have consistently demonstrated that the frequency of the A allele (encoding Arginine, Arg16) versus the G allele (encoding Glycine, Gly16) at the rs1042713 locus fluctuates drastically across disparate racial and ethnic cohorts. For instance, in the Kazakh population, the allelic frequencies of both polymorphisms diverge significantly from those observed in other Asian and European demographics, strongly indicating that distinct population genetic backgrounds fundamentally shape these polymorphic distributions [20]. These findings underscore the marked ethnic and population-specific heterogeneity of *ADRB2* polymorphisms in asthma-related research. Differences in allele frequency, genotype distribution, and associated clinical outcomes across populations indicate that the interpretation of *ADRB2* variants should be context-dependent and supported by population-specific evidence. Such heterogeneity is particularly relevant when evaluating asthma susceptibility, bronchodilator responsiveness, and exacerbation risk in different demographic settings.

In the context of bronchial asthma, the roles of the Arg16Gly and Gln27Glu polymorphisms in dictating disease susceptibility, clinical phenotypes, and pharmacological responses have been the subject of extensive, albeit occasionally controversial, research. A robust body of evidence suggests that the Arg16 allele (specifically the homozygous Arg16Arg genotype) is intricately associated with aggravated clinical manifestations and an elevated risk of asthma exacerbations. This genetic predisposition is particularly pronounced in pediatric asthmatic cohorts receiving long-acting β-2 agonist (LABA) therapy. Children harboring the Arg16 allele exhibit a markedly heightened risk of severe asthma exacerbations upon LABA administration, whereas those possessing the Gly16Gly genotype demonstrate a superior bronchodilatory response and a significantly lower incidence of exacerbations, thereby highlighting the immense potential of genotype-guided individualized therapeutic strategies [8,28].

Furthermore, comprehensive meta-analyses encompassing diverse study designs have indicated that while the baseline influence of the Arg16Gly and Gln27Glu variants on the efficacy of inhaled short-acting β-2 agonists (such as albuterol) may appear limited across general populations, profound genetic effects emerge within specific clinical subgroups. Notably, individuals with the Arg16Gly GG genotype who do not undergo bronchial provocation testing demonstrate a sub-optimal pulmonary functional response to treatment [29]. Supporting this genetic vulnerability, clinical observations in Kazakh asthmatic patients reveal that the frequency of the Arg16 allele is disproportionately elevated among individuals with uncontrolled asthma, implicating this polymorphism directly in the regulation of overall asthma control status [20]. Beyond isolated SNPs, haplotype analyses of the *ADRB2* gene provide a more comprehensive genetic perspective. The specific Arg16/Gln27 haplotype has been strongly correlated with a synergistic increase in the risk of asthma exacerbations, particularly in patients undergoing combined treatment with inhaled corticosteroids (ICS) and LABA [17]. Ultimately, the Arg16Gly and Gln27Glu polymorphisms function not merely as markers of disease susceptibility but as critical modulators of clinical severity and pharmacotherapeutic outcomes, solidifying their status as central targets in asthma genetics and precision pharmacogenomics.

To consolidate the diverse clinical implications of these major genetic variants across different ethnic populations and clinical settings, the key findings are summarized in Table 3, with study population and disease context presented as separate categories to facilitate comparison.

### 3.2. Regulatory Region SNPs and Gene-Gene Interactions

Beyond the well-characterized variants within the coding sequence, SNPs localized within the regulatory regions of the *ADRB2* gene play an indispensable role in the pathogenesis and genetic susceptibility of bronchial asthma, particularly within pediatric populations. Comprehensive genetic profiling has identified multiple polymorphic loci residing in the upstream regulatory sequences, including rs11168070, rs17108803, rs2053044, rs12654778, rs11959427, and rs2895795, which collectively govern the transcriptional dynamics of the receptor. A highly targeted case–control study comprising 143 asthmatic pediatric patients and 137 healthy controls elucidated significant disparities in both genotypic and allelic frequencies at specific loci, most notably rs2895795 (−1429T/A), rs2053044 (−1023G/A), and rs12654778 (−654G/A). Through rigorous linkage disequilibrium analysis, researchers have demonstrated that these discrete loci do not operate in isolation but rather form distinct haplotypes that profoundly influence disease risk. Specifically, the TATGCT and TATGGC haplotypes have been characterized as definitive genetic risk factors for asthma onset, whereas the AGTGCT haplotype confers a demonstrable protective effect against the disease. This strongly indicates that the combinatorial genetic background established by regulatory SNPs fundamentally dictates asthma susceptibility, rendering these haplotypes highly clinically relevant as predictive biomarkers in pediatric cohorts [31].

The pathogenic complexity of bronchial asthma extends far beyond monogenic influences and is increasingly understood as the product of epistatic interactions between *ADRB2* and key immune-regulatory genes. Among the most relevant interacting partners are IL13 and IL4, two central mediators of type 2 inflammation that contribute to allergic sensitization, airway inflammation, and IgE production. Mechanistically, IL13 and IL4 share overlapping downstream signaling pathways through receptor-mediated activation of Janus kinase (JAK) and signal transducer and activator of transcription 6 (STAT6), thereby promoting B-cell class switching toward IgE production and amplifying Th2-polarized immune responses. In parallel, *ADRB2* encodes the β2-adrenergic receptor, which not only regulates airway smooth muscle relaxation but also modulates immune-cell activity through cAMP-dependent signaling. Accordingly, the interaction between *ADRB2* variation and IL4/IL13-related loci is biologically plausible because these genes converge on airway hyperresponsiveness, inflammatory amplification, and IgE-associated asthma susceptibility.

A validated multi-locus interaction model in Han Chinese children provides empirical support for this concept. This model incorporated IL13 rs20541, IL13 rs1800925, IL4 rs2243250, and *ADRB2* rs1042713 and identified a significant four-way interaction associated with elevated cord blood IgE, a recognized early biomarker of allergic predisposition and later asthma risk [32]. Notably, IL13 rs20541 showed the strongest independent effect, whereas the remaining loci, including *ADRB2* rs1042713, appeared to exert smaller but synergistic contributions within the combined model. Children carrying the composite high-risk genotype pattern of IL13 rs20541 TT, IL13 rs1800925 CT/TT, IL4 rs2243250 TT, and *ADRB2* rs1042713 AA exhibited a markedly increased risk of elevated cord blood IgE, supporting the view that asthma susceptibility may emerge from coordinated multi-gene effects rather than from a single dominant polymorphism alone [32].

This interaction can be interpreted mechanistically at several levels. First, IL4 and IL13 enhance IgE-oriented immune polarization, while *ADRB2*-mediated signaling may influence how immune cells and airway structural cells respond to inflammatory and adrenergic stimuli. Second, variation in *ADRB2* may modify receptor responsiveness and downstream cAMP signaling, thereby altering the functional context in which Th2 cytokine pathways operate. Third, the convergence of these loci may help explain why some individuals show disproportionate allergic sensitization or bronchodilator-response heterogeneity despite carrying only modest-risk variants at individual loci. Although the precise molecular interface between *ADRB2* polymorphisms and IL4/IL13 signaling requires further experimental clarification, currently available genetic and biological evidence supports a synergistic rather than isolated model of action.

Furthermore, the pathogenesis of asthma is further shaped by the interaction of these genetic networks with environmental exposures. For example, an interaction between IL4 rs2243250 and maternal atopy has been associated with elevated cord blood IgE, suggesting that prenatal immune programming may arise from the combined effects of inherited susceptibility and the maternal atopic environment [32]. Taken together, these findings support the use of validated multi-locus interaction models to complement single-variant analysis and provide a more mechanistically grounded framework for understanding asthma susceptibility, early allergic programming, and inter-individual variation in therapeutic response.

### 3.3. Epigenetic Regulation and Environmental Interactions

While genetic sequence variations establish the foundational susceptibility to bronchial asthma, epigenetic modifications serve as the critical mechanistic bridge connecting environmental exposures to the dynamic regulation of the *ADRB2* gene expression. The complex interplay between environmental triggers, DNA methylation, and non-coding RNA networks has emerged as a paramount determinant of asthma pathogenesis and clinical control. Clinical investigations have unequivocally demonstrated that aberrant DNA methylation, particularly within the 5′-untranslated region (5′-UTR) of the *ADRB2* gene, is intimately associated with suboptimal disease management. In pediatric asthmatic cohorts, elevated systemic aluminum concentrations exhibit a profound positive correlation with the hypermethylation of the *ADRB2* 5′-UTR. Rigorous multivariate regression analyses have identified elevated blood aluminum as a potent, independent risk factor for poor asthma control, yielding a striking odds ratio of 16, while the hypermethylation state itself independently carries an odds ratio of 4.75. This compelling data delineates a clear pathogenic trajectory where environmental aluminum exposure induces targeted epigenetic silencing. The resulting hypermethylation transcriptionally represses the *ADRB2* gene, progressively diminishing the density of functional β-2 adrenergic receptors on airway smooth muscle cells, thereby attenuating bronchodilatory capacity and significantly elevating the risk of severe asthma exacerbations [33].

The epigenetic landscape of the *ADRB2* gene is further perturbed by exposure to other pervasive environmental heavy metals, including cadmium, cobalt, and lead, which operate synergistically with microRNA (miRNA) regulatory networks to exacerbate airway pathology. Case–control studies involving adult asthmatic patients have revealed that elevated blood concentrations of cadmium and cobalt are significantly correlated with an augmented methylation status at the *ADRB2* 5′-UTR. Concurrently, these heavy metal burdens precipitate a marked downregulation of miRNA-146a, a critical post-transcriptional regulator heavily implicated in the maintenance of immune and inflammatory homeostasis. The inverse relationship between heavy metal-induced *ADRB2* hypermethylation and diminished miRNA-146a expression elucidates a highly complex, multi-layered epigenetic disruption. The suppression of miRNA-146a not only deregulates local immune responses, leading to an intensified infiltration of inflammatory cells, but also acts in concert with DNA hypermethylation to comprehensively impair β-2 receptor functionality. This dual epigenetic assault profoundly intensifies airway hyperresponsiveness and accelerates the clinical onset of asthma, positioning the *ADRB2* methylation status and miRNA-146a levels as highly valuable predictive biomarkers for environmental-induced respiratory diseases [34].

Crucially, the inherent plasticity of epigenetic modifications presents a highly promising frontier for novel therapeutic interventions. Unlike immutable genetic polymorphisms, DNA methylation patterns induced by heavy metal toxicity exhibit a degree of reversibility. Preclinical investigations have demonstrated that the application of DNA methyltransferase inhibitors, such as 5-azacytidine, can effectively recalibrate aberrant methylation profiles. By inhibiting hypermethylation, these pharmacological agents can restore normative *ADRB2* transcriptional activity and rescue receptor function, thereby offering a highly innovative, epigenetically targeted strategy for asthma management [18].

In addition to environmental exposures, therapeutic exposure itself may represent a dynamic modifier of *ADRB2* regulation. Prolonged or repeated β2-agonist treatment has long been associated with receptor desensitization and diminished bronchodilator responsiveness, and emerging evidence suggests that chronic treatment may also contribute to transcriptionally suppressive or epigenetically mediated changes in *ADRB2* expression. Such treatment-related alterations could reduce receptor abundance over time and may interact with pre-existing genetic polymorphisms, thereby further complicating the relationship between genotype and clinical drug response. This point is particularly important in the interpretation of long-term pharmacotherapy, because the functional consequences of chronic β2-agonist exposure may not be fully captured by baseline genotyping alone.

Beyond the direct toxicity of heavy metals, the epigenetic regulation of *ADRB2* is also sensitive to broader metabolic, lifestyle, and host-related factors, including obesity, physical activity, and inherited susceptibilities. Crucially, the inherent plasticity of epigenetic modifications presents a highly promising frontier for novel therapeutic interventions. Unlike immutable genetic polymorphisms, DNA methylation patterns induced by heavy metal toxicity exhibit a degree of reversibility. Preclinical investigations have demonstrated that the application of DNA methyltransferase inhibitors, such as 5-azacytidine, can effectively recalibrate aberrant methylation profiles. By inhibiting hypermethylation, these pharmacological agents can restore normative *ADRB2* transcriptional activity and rescue receptor function, thereby offering a highly innovative, epigenetically targeted strategy for asthma management [18]. Recent transcriptomic and epigenetic profiling indicates that regular physical activity and the minimization of sedentary behaviors in pediatric and adolescent populations significantly optimize the epigenetic status of asthma-related genes. These lifestyle modifications actively modulate systemic immune-inflammatory responses, thereby conferring a protective epigenetic phenotype against asthma pathogenesis [35]. Furthermore, an established maternal history of atopy acts as a foundational genetic predisposition that, when coupled with gestational or early-life environmental exposures, can program the fetal epigenome. This intricate gene-environment interaction dynamically alters the baseline expression state of the *ADRB2* gene, fundamentally shaping the offspring’s lifelong susceptibility to allergic airway diseases.

To synthesize the multifaceted interactions among environmental exposures, host-related modifiers, pharmacologic interventions, and epigenetic mechanisms affecting *ADRB2* function, these relationships are detailed in Table 4.

## 4. Clinical Applications: Pharmacogenomics and Precision Medicine

### 4.1. Impact of Polymorphisms on Bronchodilator Efficacy and Adverse Events

Bronchodilators, specifically short-acting and long-acting β-2 agonists (SABAs and LABAs), represent the pharmacological cornerstone for both the acute relief and chronic management of bronchial asthma. However, clinical trajectories consistently reveal profound inter-individual heterogeneity in therapeutic efficacy, a phenomenon deeply rooted in the pharmacogenomic landscape of the *ADRB2* gene. The functional polymorphisms Arg16Gly and Gln27Glu fundamentally govern the structural dynamics of the β-2 adrenergic receptor and its subsequent responsiveness to exogenous agonists. Large-scale pharmacogenomic evaluations, such as the comprehensive analysis conducted by the PiCA consortium encompassing 5903 asthmatic patients, have robustly demonstrated that specific allelic combinations dictate disparate treatment outcomes. Patients harboring the Arg16/Gln27 haplotype and receiving concurrent therapy with inhaled corticosteroids (ICS) and LABAs exhibit a significantly escalated risk of asthma exacerbations. This deleterious clinical trajectory suggests that this specific haplotype compromises the sustained bronchodilatory efficacy of LABAs, thereby increasing the probability of disease deterioration. Conversely, individuals possessing the Gly16/Glu27 haplotype demonstrate a markedly lower risk of exacerbations, underscoring the critical necessity of utilizing haplotypic profiling to stratify patient risk and optimize individualized therapeutic regimens [17].

In the context of acute symptom relief, the therapeutic efficacy of SABAs, most notably albuterol, is similarly modulated by these critical genetic variations. The A to G nucleotide transition at position 46 of the *ADRB2* gene, which precipitates the Arg16Gly substitution, critically alters the receptor’s pharmacological sensitivity. Advanced molecular diagnostic platforms utilizing real-time allele-specific polymerase chain reaction (AS-PCR) have facilitated high-throughput genotypic stratification, revealing that patients carrying the Arg16 allele exhibit a superior, highly responsive bronchodilatory reaction to albuterol administration. In stark contrast, individuals possessing the Gly16 allele frequently present with attenuated pharmacological responses and sub-optimal airway relaxation. These clinical observations strongly advocate for the integration of the Arg16Gly polymorphism as a highly reliable predictive biomarker for SABA efficacy [10]. Furthermore, the Gln27Glu polymorphism also exerts considerable influence over drug responsiveness, primarily by modulating the complex intracellular kinetics of receptor desensitization and endocytosis, thereby dictating the duration and stability of the bronchodilatory effect.

The pharmacogenomic influence of *ADRB2* polymorphisms does not operate in a biological vacuum but is intricately modulated by demographic and anthropometric variables. Comprehensive clinical investigations involving expansive patient cohorts have identified a profound gene-age interaction, demonstrating that the modulatory effect of *ADRB2* polymorphisms on bronchodilator responsiveness fluctuates significantly across different age demographics. This age-dependent genetic penetrance implies that pediatric, adult, and geriatric asthmatic populations may require fundamentally distinct, genotype-calibrated therapeutic strategies to achieve optimal clinical outcomes [36]. Additionally, the pervasive influence of ethnic and racial genetic divergence further complicates the global applicability of standardized pharmacological protocols. Extensive sequencing analyses targeting SNPs within the *ADRB2* and ADCY9 genes in diverse Latin American populations, such as those in Brazil, have elucidated stark disparities in the distribution of these critical genetic variants across different racial groups. This profound population stratification provides a definitive molecular rationale for the widely observed discrepancies in drug responsiveness across diverse global demographics, thereby reinforcing the imperative for population-specific pharmacogenomic research [37].

Beyond modulating therapeutic efficacy, *ADRB2* polymorphisms act as pivotal determinants of drug-induced adverse events and the insidious development of pharmacological tolerance. A critical clinical manifestation of this genetic vulnerability is observed in patients afflicted with aspirin-exacerbated respiratory disease (AERD). In these highly susceptible individuals, the administration of aspirin or other nonsteroidal anti-inflammatory drugs (NSAIDs) provokes severe, sometimes life-threatening bronchoconstriction and a rapid deterioration of clinical symptoms. In-depth genetic analyses have firmly established a robust correlation between specific *ADRB2* polymorphisms and the pathogenesis of AERD. These genetic sequence variations fundamentally alter the baseline functional integrity and compensatory capacity of the β-2 adrenergic receptor, thereby pathologically amplifying airway hyperresponsiveness to specific pharmacological triggers. This profound genetic heterogeneity significantly elevates the risk profile for severe adverse drug reactions, rendering the clinical management of such patients exceptionally challenging. By leveraging high-resolution molecular polymorphism analysis, clinicians can accurately identify these high-risk patient subpopulations pre-emptively, thereby facilitating the implementation of individualized, risk-averse pharmacological strategies that minimize the incidence of severe iatrogenic events [38].

Furthermore, the progressive development of drug tolerance, a major obstacle in chronic asthma management, is intimately linked to the underlying *ADRB2* genotype. Patients harboring specific genetic variants frequently exhibit an accelerated decline in receptor sensitivity following prolonged exposure to β-2 agonists, a physiological adaptation that directly culminates in therapeutic recalcitrance and diminished clinical efficacy over time. This genotype-driven tolerance is particularly detrimental in complex disease phenotypes such as AERD, where the intersection of heightened inflammatory tone and rapid receptor desensitization renders standard therapeutic algorithms largely ineffective. Therefore, the rigorous profiling of *ADRB2* polymorphisms is not solely diagnostic but constitutes an indispensable predictive tool for anticipating individualized patterns of drug tolerance, ultimately enabling the continuous optimization of therapeutic regimens to ensure sustained clinical efficacy and maximized patient safety [38].

### 4.2. Potential Clinical Applications of Genotype-Guided Therapy and Economic Evaluation

The β2-adrenergic receptor encoded by *ADRB2* is an important determinant of bronchodilator responsiveness and has therefore become a major focus of asthma pharmacogenomics. Common variants, particularly Arg16Gly (rs1042713), may contribute to inter-individual differences in treatment response and have prompted growing interest in genotype-informed prescribing. However, the clinical translation of this approach should currently be interpreted with caution. Although several studies, especially in pediatric populations, suggest that *ADRB2* genotyping may help identify subgroups with differential responses to LABA-containing regimens, major contemporary asthma guidelines do not currently recommend routine pharmacogenetic testing before LABA prescription. Current treatment algorithms remain based primarily on symptom control, exacerbation risk, clinical phenotype, inhaler technique, treatment accessibility, and other patient-specific factors. Therefore, *ADRB2* genotype-guided therapy should presently be regarded as a promising but still exploratory precision-medicine strategy rather than an established component of standard asthma care.

**Figure 2:**
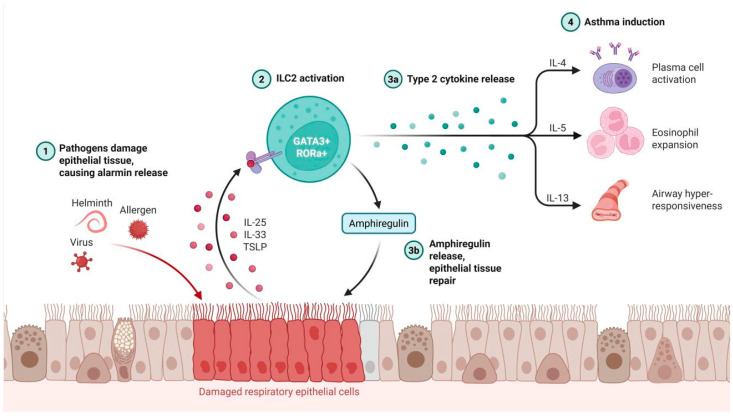
Gene-Environment-Epigenetic Interactions and a Proposed Framework for Genotype-Informed Asthma Management.

The Arg16Gly polymorphism exerts a substantial influence on baseline β-2 receptor functionality and its subsequent pharmacological responsiveness. Specifically, patients harboring the Arg16 variant (the A allele) frequently exhibit sub-optimal clinical responses to conventional LABA therapy, a molecular phenotype that is inherently associated with a markedly elevated risk of severe asthma exacerbations. Compelling clinical evidence derived from an individual participant data meta-analysis of two independent, randomized controlled trials (the PUFFIN and PACT trials) has definitively corroborated the clinical superiority of genotype-guided treatment adjustments. This comprehensive evaluation demonstrated that prescribing LABA step-up therapy exclusively to children possessing the homozygous Gly16/Gly16 genotype, while simultaneously redirecting Arg16 carriers toward alternative therapeutic modalities (such as doubled-dose ICS or the addition of leukotriene receptor antagonists like montelukast), precipitates a statistically significant reduction in overall exacerbation rates [8]. Furthermore, parallel pharmacogenomic investigations emphasize the critical association between the specific *ADRB2* genotype and the highly variable responsiveness to short-acting inhaled β-2 agonists, thereby establishing a robust, evidence-based scientific foundation for precision pharmacotherapy [10], as shown in Figure 2. Moreover, because long-term β2-agonist exposure may itself alter *ADRB2* expression and receptor responsiveness through adaptive and potentially epigenetically mediated mechanisms, the predictive value of genotyping may decrease over the course of chronic therapy.

Importantly, genotype-based testing captures inherited DNA sequence variation but does not directly reflect the functional state of the receptor at the time of treatment. As discussed in the epigenetic section, *ADRB2* expression and β2-agonist responsiveness can be substantially modified by DNA methylation and other epigenetic mechanisms, which may alter receptor abundance and downstream signaling without changing the underlying genotype. Therefore, patients with the same *ADRB2* genotype may still exhibit different levels of receptor expression and different clinical responses to bronchodilator therapy. This limitation should be explicitly considered when interpreting pharmacogenetic results, because genotype alone may not fully predict treatment efficacy unless it is integrated with epigenetic status and broader clinical context.

Encouraging findings from genotype-stratified studies suggest that *ADRB2* variation may eventually contribute to more individualized selection of controller and reliever therapies. Nevertheless, the current evidence remains insufficient for broad implementation in routine practice because the magnitude and consistency of genotype-treatment interactions vary across age groups, study designs, and clinical settings. In addition, many of the reported benefits have been derived from selected cohorts and economic models rather than universal real-world adoption. Accordingly, before *ADRB2*-guided prescribing can be incorporated into standard clinical algorithms, further prospective multicenter trials, external validation in diverse populations, assay standardization, and formal evaluation by guideline panels are still required.

The unpredictable nature of acute asthma exacerbations presents a formidable challenge in chronic respiratory disease management; however, genotype-guided therapeutic strategies significantly mitigate this risk by precisely matching the pharmacological intervention to the patient’s intrinsic molecular profile. Traditional LABA administration in patients harboring the Arg16 allele paradoxically exacerbates the risk of clinical deterioration, whereas the immediate implementation of genotype-directed alternative therapies neutralizes this heightened genetic vulnerability [8]. Extensive multicenter validations have further cemented the applicability and profound clinical utility of this precision strategy across diverse patient demographics, demonstrating its immense capacity to facilitate the early identification of high-risk asthmatic subpopulations [39]. Empowered by rapid genetic screening, physicians can proactively recalibrate treatment trajectories, thereby comprehensively elevating the standard of asthma control, significantly reducing the burden of emergency department visits and inpatient hospitalizations, and ultimately optimizing the patient’s long-term quality of life.

Despite these encouraging findings, the real-world implementation of *ADRB2*-guided prescribing remains constrained by several practical barriers. First, the availability of pharmacogenetic testing is still highly uneven across healthcare systems, and access may be limited by local laboratory capacity, turnaround time, and the absence of routine testing pathways in respiratory practice. Second, the economic value of genotyping is likely to vary substantially across countries and clinical settings, because testing costs, reimbursement policies, drug pricing, and the baseline risk of exacerbation differ widely between healthcare systems. Therefore, cost-effectiveness estimates derived from specific national models should not be directly generalized to all clinical environments. Third, even when genotyping is technically available, broader implementation requires standardized assay methods, consistent interpretation of variants, integration into clinical decision-support systems, and clear guidance regarding which patient subgroups are most likely to benefit. These considerations indicate that the translational pathway from pharmacogenomic association to routine asthma management remains incomplete.

Beyond direct financial metrics, *ADRB2* genotyping may help optimize healthcare resource utilization in selected settings, but its broader implementation remains dependent on local infrastructure, reimbursement policies, and clinical workflow integration. By accurately predicting an individual’s distinct responsiveness to β-2 agonists, healthcare providers can unequivocally eliminate the prescription of physiologically ineffective medications, thereby preventing medication-induced adverse reactions and circumventing the exorbitant costs associated with acute hospital admissions. Concurrently, the continuous refinement of molecular diagnostic technologies, such as the deployment of real-time allele-specific PCR, has drastically accelerated the speed, enhanced the analytical precision, and reduced the operational costs of genetic sequencing [10]. This technological maturation provides an accessible and scalable logistical foundation for the widespread clinical adoption of genotype-guided asthma therapy, promising a future characterized by enhanced health outcomes and streamlined medical expenditures.

As a paradigmatic representation of precision medicine, *ADRB2* genotype-guided therapy has garnered unprecedented attention within the clinical management of complex respiratory syndromes. The profound pharmacological impact of the Arg16Gly (rs1042713) and Gln27Glu (rs1042714) polymorphisms extends beyond β-2 agonists (such as albuterol) to also include the varied therapeutic efficacy and safety profiles of β-blockers. Clinical outcome data consistently highlight that the Arg16 allele predisposes pediatric patients to an elevated risk of LABA-induced exacerbations, whereas the Gly16Gly genotype correlates with superior therapeutic responsiveness, findings that are heavily supported by the cost-effective interventions modeled in the aforementioned PUFFIN and PACT trials [8,17,28,39]. With the increasing accessibility of sequencing technologies, *ADRB2* genotyping has attracted interest not only in asthma pharmacogenomics but also in related airway diseases such as COPD [10,40,41,42,43]; however, its most clinically developed applications remain within the context of bronchodilator responsiveness and treatment stratification in obstructive airway disease. Nevertheless, the comprehensive integration of these molecular diagnostics into routine clinical practice is severely impeded by formidable translational hurdles, primarily concerning the international standardization of sequencing protocols, the complex interpretation of variant data, and the urgent necessity to construct robust clinical decision support systems.

Finally, the clinical translation of *ADRB2*-guided pharmacotherapy is inextricably linked to a complex matrix of ethical, legal, and sociopolitical considerations. The inherent sensitivity of individualized genomic data necessitates the implementation of stringent privacy protocols during the acquisition, storage, and clinical utilization of genetic profiles to neutralize the profound risks of data breaches and subsequent genetic discrimination. Prior to initiating molecular diagnostics, clinicians are ethically obligated to secure comprehensive informed consent, meticulously elucidating the explicit clinical objectives, the potential psychological ramifications, and the predictive limitations of the genomic results [28]. Furthermore, within the immediate clinical environment, genotype-guided interventions precipitate critical ethical dilemmas, most notably regarding the equitable distribution of finite healthcare resources and the potential precipitation of profound psychological distress. Patients receiving unfavorable genetic prognostications may experience acute anxiety; thus, the integration of comprehensive genetic counseling services, operated by highly trained medical personnel capable of deciphering complex genotypic implications, is absolutely paramount [44]. While initial economic evaluations confirm the immense cost-effectiveness of *ADRB2* genotyping, the financial feasibility of deploying advanced molecular diagnostics in resource-constrained environments remains a critical topic of ongoing debate [39]. Ultimately, the widespread clinical success of *ADRB2* genotype-guided therapy relies entirely upon a harmonized, multi-disciplinary approach that addresses technical feasibility, enforces rigorous ethical standards, and establishes comprehensive policy frameworks to actualize the profound societal benefits of precision medicine.

### 4.3. Advances in ADRB2-Targeted Gene Delivery Systems

In recent years, the paradigm of *ADRB2*-targeted therapy has progressively expanded beyond traditional small-molecule pharmacology to encompass sophisticated gene therapy modalities for bronchial asthma and related airway pathologies. The fundamental prerequisite for the successful clinical translation of gene regulation strategies lies in the development of highly efficient and exceptionally specific gene delivery systems. Historically, conventional gene vectors have been critically limited by suboptimal tissue tropism and inadequate transfection efficiencies, severely impeding their therapeutic utility in pulmonary medicine. To circumvent these formidable biological barriers, researchers have engineered a variety of novel, precision-targeted delivery platforms. Among these cutting-edge innovations, polyethyleneimine-modified isoprenaline (PEI-isoprenaline) has emerged as a highly promising, ligand-conjugated nanoparticle carrier, exhibiting both outstanding biocompatibility and unprecedented targeted delivery capabilities [45].

The mechanistic ingenuity of the PEI-isoprenaline system relies on the strategic incorporation of isoprenaline, a potent β-adrenergic agonist, onto the polymeric backbone of PEI. This structural modification facilitates the highly specific molecular recognition and subsequent binding of the nanoparticle complex exclusively to cells expressing the *ADRB2*. Consequently, this targeted tropism drastically amplifies the intracellular delivery efficiency of nucleic acid therapeutics, such as small interfering RNA (siRNA). Comprehensive in vitro cytotoxicity assays utilizing the human bronchial epithelial cell line Beas-2B have unequivocally demonstrated that both the naked PEI-isoprenaline vector and its siRNA-loaded complexes possess a highly favorable safety profile, thereby substantiating their suitability for subsequent clinical translation [45]. Furthermore, rigorous in vivo evaluations conducted in murine asthma models have validated the exceptional performance of this platform. The PEI-isoprenaline system successfully delivered therapeutic siRNA specifically to *ADRB2*-expressing cell populations within the bronchoalveolar lavage fluid and the pulmonary parenchyma, achieving a remarkable transfection efficiency of 80%, which vastly eclipses the mere 20% efficiency observed with conventional, unmodified PEI 2K vectors [45]. Despite these promising findings, several issues must be addressed before PEI-isoprenaline or related *ADRB2*-targeted carriers can be translated into clinical application. First, the currently available safety data are largely limited to in vitro assays and short-term murine experiments; therefore, long-term toxicity, repeated-dose tolerance, immunogenicity, off-target biodistribution, and the consequences of chronic airway exposure remain insufficiently defined. This is particularly important for pulmonary gene delivery, because inhaled nanoparticles must overcome mucus entrapment, mucociliary clearance, alveolar macrophage uptake, and variable deposition across diseased airways. Second, although ligand-modified PEI improves cell-specific targeting relative to unmodified polymeric carriers, scalability, formulation stability, aerosol performance, and reproducible manufacturing under clinically relevant conditions still require further optimization. Third, in comparison with alternative delivery platforms, viral vectors generally provide high transduction efficiency but are constrained by immunogenicity and payload-related limitations, whereas non-viral systems such as polymeric or lipid-based nanoparticles may offer improved safety and formulation flexibility but often show lower or less durable gene expression in vivo. Therefore, PEI-isoprenaline should currently be viewed as a promising targeted non-viral platform whose translational value will depend on further comparative and long-term validation.

The targeted delivery mediated by the PEI-isoprenaline system provides a highly precise therapeutic avenue for intervening in the complex pathogenesis of asthma, particularly concerning the phenomenon of airway remodeling. Airway remodeling, characterized by irreversible structural alterations and fibrotic deposition within the respiratory tract, constitutes a primary driver of sustained airflow obstruction and progressive functional decline in chronic asthma. Given that *ADRB2* functions as a master regulator of both airway smooth muscle relaxation and localized inflammatory cascades, targeted genetic modulation of this receptor and its associated downstream effectors offers a potent strategy for disrupting these pathogenic structural changes. In established murine models, the PEI-isoprenaline-mediated delivery of siRNA specifically engineered to downregulate the critical pathogenic gene arginase-1 yielded profound therapeutic outcomes. This targeted transcriptional suppression significantly attenuated the localized infiltration of pro-inflammatory cells, drastically reduced airway fibrotic deposition, and effectively inhibited the overarching progression of pathological airway remodeling, ultimately ameliorating airway hyperresponsiveness [45].

Overall, the PEI-isoprenaline delivery system represents an intriguing advance in *ADRB2*-targeted respiratory gene therapy and provides proof of concept that ligand-directed non-viral carriers can improve pulmonary siRNA delivery in experimental asthma. The currently available data support its potential to reduce airway inflammation and remodeling in preclinical models; however, its clinical relevance remains to be established through longer-term safety studies, repeated-dose assessments, optimization for inhaled delivery, and direct comparison with alternative viral and non-viral platforms. Future work should therefore focus not only on therapeutic efficacy but also on translational feasibility, including formulation robustness, airway deposition, mucus penetration, manufacturability, and reproducibility across disease settings.

## 5. Emerging Concepts and Future Perspectives

### 5.1. Shared Genetic Mechanisms in Airway Diseases

The β-2 adrenergic receptor, encoded by the *ADRB2* gene, plays a quintessential role in modulating airway smooth muscle relaxation and local inflammatory cascades. Recent large-scale genomic investigations have progressively revealed that the genetic polymorphisms of *ADRB2* dictate not only the clinical trajectory and pharmacological responsiveness of bronchial asthma but also exhibit a profound genetic correlation with COPD. Comprehensive comparative analyses demonstrate that *ADRB2*, alongside other critical immune-regulatory genes, confers overlapping risk profiles across both asthma and COPD patient cohorts, strongly implying that these two prevalent respiratory disorders share a foundational pleiotropic genetic architecture [46]. Specifically, the differential distribution of the *ADRB2* rs1042713 (Arg16Gly) allele is highly correlated with the variability in bronchodilator responsiveness among asthmatic individuals, while simultaneously demonstrating a significant association with progressive lung function decline and specific inflammatory phenotypes in patients afflicted with COPD [29,47]. Furthermore, pervasive polymorphisms within the FCER2 gene have been shown to modulate cross-disease susceptibility, indicating that these genetic variations govern common pathophysiological pathways characterized by chronic airway inflammation and aberrant immune mechanisms [46]. This substantial genetic overlap provides robust molecular validation for the “Dutch hypothesis”, a classical theoretical framework positing that asthma and COPD, despite presenting divergent clinical phenotypes and distinct histological pathologies, are fundamentally rooted in a shared continuum of genetic and environmental interactions. Consequently, the implementation of comprehensive, pan-airway genetic screening panels is absolutely imperative for deciphering the immense complexity of these overlapping respiratory syndromes.

Because bronchial asthma manifests as a highly complex, chronic inflammatory disease, its pathogenesis is dictated by the intricate interplay of polygenic networks and pervasive environmental stimuli. In recent years, the conceptualization and rigorous validation of advanced multi-gene models have provided a transformative perspective for unraveling the profound genetic heterogeneity inherent in asthma. For instance, a sophisticated four-gene predictive model encompassing *ADRB2* rs1042713, IL4 rs2243250, FCER1B rs569108, and IL13 rs20541 has been validated within Chinese Han pediatric populations, demonstrating exceptional prognostic accuracy in evaluating cumulative asthma risk [48,49]. This multi-locus framework not only highlights the powerful epistatic synergies existing between disparate genetic loci but also quantitatively reflects the multidimensional influence of the inherited genetic background on individualized disease susceptibility. Notably, the predictive efficacy of this polygenic model fluctuates significantly across different ethnic cohorts, thereby reiterating the critical necessity of accounting for deep racial and genetic heterogeneity when mapping the precise genetic determinants of asthma [48].

Beyond merely assessing disease risk, multi-gene models significantly augment the clinical ability to predict inter-individual variations in bronchodilator responsiveness. While isolated *ADRB2* polymorphisms exhibit marked individual variability in their modulation of albuterol efficacy, integrating these variants with the polymorphic profiles of concurrently active immune-regulatory genes facilitates a highly precise anticipation of therapeutic outcomes [10,47]. By synthesizing massive datasets of multi-locus genetic information, these advanced polygenic models vastly enhance the analytical resolution of asthma genetics. They supply a formidable theoretical foundation for the formulation of genotype-guided, precision-targeted clinical strategies, ultimately propelling the clinical translation of personalized respiratory medicine.

To delineate the shared genetic overlaps and multi-gene synergies governing these complex airway diseases, the core genetic models and their corresponding clinical implications are summarized in Table 5.

### 5.2. Future Research Directions

The extensive body of contemporary research unequivocally elucidates that the genetic polymorphisms of the *ADRB2* gene occupy a central, highly deterministic position within the complex biological networks governing the pathogenesis, phenotypic expression, and pharmacological responsiveness of bronchial asthma. These specific genetic variations fundamentally alter the structural conformation and functional plasticity of the β-2 adrenergic receptor, thereby directly dictating the highly variable clinical sensitivity and tolerance observed toward widely administered bronchodilators. This profound mechanistic understanding establishes an irrefutable genetic foundation for comprehending inter-individual disease heterogeneity, ultimately serving as the robust theoretical rationale for the immediate clinical implementation of genotype-calibrated therapeutic regimens.

However, the progressive trajectory of bronchial asthma is rarely dictated by isolated monogenic influences; rather, it represents the cumulative culmination of highly intricate, multi-factorial interactions. The profound modulatory impact of epigenetic regulation, operating in tandem with pervasive environmental exposures, has garnered unprecedented scientific focus. This emerging paradigm not only exponentially enriches our contemporary understanding of asthma pathogenesis but also underscores the profound complexity of dynamic gene-environment interactions in determining disease severity. Given the inherent methodological disparities across existing studies concerning specific epigenetic modifications and environmental triggers, it is imperative that future investigative efforts adopt a highly cautious, multidimensional, and rigorously integrated analytical approach. Such comprehensive methodologies are essential to circumvent biased interpretations and to accurately delineate the specific regulatory pathways governing *ADRB2* expression within the pulmonary microenvironment.

From a translational perspective, individualized therapeutic strategies directed by *ADRB2* genotypic profiling exemplify the pinnacle of precision respiratory medicine, demonstrating immense clinical and health economic potential. By strategically optimizing pharmacological selections and titrating dosages based on an individual’s distinct genetic architecture, clinicians can effectively precipitate a marked reduction in the incidence of acute asthma exacerbations while simultaneously mitigating the extensive economic burden associated with healthcare resource misallocation. Nevertheless, the widespread implementation of these precision strategies continues to face substantial translational hurdles, including uneven access to testing, variability in reimbursement and cost-effectiveness across healthcare systems, lack of assay and interpretation standardization, and the absence of universally adopted guideline frameworks for routine clinical use. Consequently, future research trajectories must prioritize the bioengineering of highly streamlined, cost-effective molecular diagnostic platforms and vigorously foster cross-disciplinary collaborations to seamlessly integrate genomic intelligence into routine clinical decision-making frameworks.

Looking forward, the systematic integration of cutting-edge discoveries across the disciplines of classical genetics, high-resolution epigenomics, and environmental toxicology will be absolutely paramount for comprehensively mapping the sophisticated regulatory networks of the *ADRB2* gene. This multidisciplinary synthesis will not only dismantle existing pharmacological bottlenecks but also pioneer revolutionary conceptual frameworks for the early predictive screening, prophylactic prevention, and highly targeted intervention of allergic airway diseases. Furthermore, the rapid maturation of revolutionary gene delivery and editing technologies, particularly CRISPR-Cas9 systems and advanced nanocarrier platforms, presents an unprecedented epoch of opportunity for direct, *ADRB2*-targeted genetic therapeutics. These sophisticated bioengineering technologies hold the transformative potential to achieve exceptionally precise and fundamentally safe genetic modulation, thereby dramatically elevating the therapeutic efficacy and long-term quality of life for asthmatic patients. Ultimately, as *ADRB2*-centric research continues to accelerate, sustaining rigorous, multi-level interdisciplinary alliances will be essential to translate these profound molecular insights into tangible, widespread health improvements for the global asthmatic population.

## 6. Conclusions

In conclusion, the *ADRB2* constitutes a critical molecular cornerstone governing the pathogenesis, phenotypic heterogeneity, and pharmacological responsiveness of bronchial asthma. Extensive genetic and molecular profiling has unequivocally established that functional polymorphisms within this locus actively dictate the structural dynamics of the receptor, thereby serving as the fundamental genetic basis for inter-individual variability in bronchodilator efficacy and providing a robust theoretical rationale for individualized clinical interventions. However, the progression of asthma is a highly complex, multi-factorial process heavily influenced by the intricate interplay between the inherited genetic sequence and the external environment. The integration of epigenetic modifications, specifically environmentally induced DNA methylation and microRNA dysregulation, highlights a multidimensional regulatory network that requires comprehensive, multi-level analysis to fully decipher the spatiotemporal mechanisms of *ADRB2* expression. Accordingly, *ADRB2* genotyping alone should not be interpreted as a complete surrogate for receptor functional status, because epigenetic regulation may substantially modify treatment responsiveness beyond the inherited DNA sequence.

The paradigm of *ADRB2* genotype-guided individualized therapy represents a transformative, highly promising leap toward precision respiratory medicine. By strategically aligning pharmacological interventions and dosage adjustments with a patient’s distinct genetic architecture, clinicians can effectively optimize therapeutic efficacy, significantly diminish the risk of severe asthma exacerbations, and alleviate the overarching economic burden on global healthcare systems. To fully realize this clinical potential, future medical frameworks must overcome existing translational challenges by developing highly accessible, cost-effective genetic diagnostic platforms and enhancing the genomic interpretive capacity of frontline clinicians. Furthermore, as emerging bioengineering technologies, such as CRISPR-based gene editing and advanced nanocarrier delivery systems, continue to mature, they present unprecedented opportunities for highly targeted, safe, and precise genetic modulation. Ultimately, fostering continuous, multi-disciplinary cooperation will be absolutely essential to translate the profound molecular insights of *ADRB2* research into widespread, tangible clinical applications, thereby achieving true precision management and fundamentally improving the lifelong health outcomes of the global asthmatic population.

## Figures and Tables

**Table 1:** Key Structural Domains and Amino Acid Residues of the β-2 Adrenergic Receptor (β2AR) and Their Biological Functions.

Structural Domain/ Residue	Location	Primary Biological Function/Mechanism	Reference
TM1 to TM7	Transmembrane core	Forms the canonical seven-transmembrane architecture essential for receptor signaling and structure.	[10,11]
TM6	Transmembrane core	Undergoes a 6 to 14 Angstrom outward displacement essential for G protein coupling and activation.	[12]
Intracellular C-terminus	Intracellular	Serves as a critical regulatory region for post-translational modifications (phosphorylation, ubiquitination).	[11]
F193	Extracellular Loop 2 (ECL2)	Coordinates β-arrestin binding and regulates biased signaling pathways.	[13]
D113, N312, S203, S204, S207	Orthosteric binding pocket	Forms a critical hydrogen bond network for the high-affinity binding of endogenous and exogenous agonists.	[14]

**Table 2:** Summary of Signal Transduction Pathways and Transcriptional Regulation of *ADRB2*.

Regulatory Mechanism/ Pathway	Core Molecular Mediators	Primary Physiological/ Cellular Outcome	Reference
Canonical Gs Signaling	Gs protein, Adenylyl Cyclase, cAMP, PKA	Inhibits myosin light chain kinase (MLCK), leading to robust airway smooth muscle relaxation.	[10,15]
Biased Signaling	Β-arrestin, MAPK cascades	Mediates receptor internalization, desensitization, and regulates cellular proliferation and apoptosis.	[13,16]
Conformational Dynamics	Transmembrane helix VII (TM VII)	Spontaneous fluctuation dictates signal intensity and bias kinetics even in unliganded states.	[16]
Post-Translational Modification	Ubiquitin, Phosphorylation (C-tail)	Modulates β-arrestin affinity, fine-tuning signal duration and intracellular trafficking.	[11]
Transcriptional Regulation	E2F1 Transcription Factor	Directly binds the 5′ regulatory region to amplify *ADRB2* mRNA transcription and protein synthesis.	[19]
Genetic and Epigenetic Impact	SNPs (Arg16Gly, Gln27Glu), DNA Methylation	Modifies receptor functional capacity, basal expression levels, and clinical bronchodilator efficacy.	[10,17,18]

**Table 3:** Clinical Implications of Major *ADRB2* Polymorphisms Across Different Populations and Clinical Contexts.

Polymorphism (SNP)	Allele/ Genotype/ Haplotype	Clinical Association or Phenotypic Impact	Study Population/ Ethnicity	Disease/Clinical Context	Reference
Arg16Gly (rs1042713)	Variable allele frequencies	Significant divergence from other Asian and European populations; Arg16 linked to uncontrolled asthma	Kazakh	Bronchial asthma	[20]
Arg16Gly (rs1042713)	GA genotype	Significantly associated with disease pathogenesis	Indian	Primary open-angle glaucoma (POAG)	[30]
Gln27Glu (rs1042714)	G allele	Acts as a protective factor against disease manifestation	Saudi Arabian	Polycystic ovary syndrome (PCOS)	[7]
Arg16Gly (rs1042713)	Arg16 allele/Arg16Arg genotype	Increased risk of severe exacerbations during LABA therapy	Pediatric patients	Bronchial asthma	[8,28]
Arg16Gly (rs1042713)	Gly16Gly genotype	Better bronchodilator response and fewer exacerbations during LABA therapy	Pediatric patients	Bronchial asthma	[8,28]
Arg16Gly (rs1042713)	GG genotype	Sub-optimal pulmonary functional response to short-acting β2-agonists in specific subgroups	Mixed or general asthma cohorts	Bronchial asthma	[29]
Combined haplotype	Arg16/Gln27 haplotype	Increased risk of exacerbations during combined ICS/LABA therapy	Mixed or general asthma cohorts	Bronchial asthma	[17]

**Table 4:** Epigenetic Regulators, Environmental Triggers, and Their Clinical Impacts on *ADRB2* Function.

Exposure/Regulator/ Intervention	Category	Epigenetic Mechanism	Molecular Consequence	Clinical Impact on Asthma	Reference
Aluminum exposure	Environmental exposure	Hypermethylation of the *ADRB2* 5′-UTR	Represses *ADRB2* transcription and reduces receptor density	Associated with poor asthma control and elevated exacerbation risk	[33]
Cadmium and cobalt exposure	Environmental exposure	Hypermethylation of 5′-UTR and suppression of miRNA-146a	Impairs β2 receptor functionality and immune homeostasis	Intensifies airway hyperresponsiveness and asthma onset	[34]
DNA methyltransferase inhibitors	Pharmacologic intervention	Inhibition of hypermethylation	Restores *ADRB2* transcriptional activity in preclinical models	Potential therapeutic strategy for reversing epigenetically mediated receptor dysfunction	[18]
Physical activity and lifestyle	Host/lifestyle modifier	Modulation of systemic epigenetic profiles	Optimizes asthma-related gene transcription and mitigates inflammation	Protective epigenetic phenotype	[35]
Maternal atopy + environmental toxins	Gene-environment modifier	Fetal epigenome programming	Alters baseline *ADRB2* expression	Elevates lifelong susceptibility to allergic airway disease	[35]

**Table 5:** Shared Genetic Mechanisms and Polygenic Models in Asthma and COPD.

Genetic Locus/Polygenic Model	Target Disease (s)	Primary Pathophysiological Role and Clinical Association	Reference
*ADRB2* rs1042713 (Arg16Gly)	Asthma and COPD	Modulates bronchodilator responsiveness (Asthma); it strongly correlates with lung function decline and inflammatory phenotypes (COPD).	[29,47]
FCER2 Polymorphisms	Asthma and COPD	Modulates cross-disease susceptibility by governing common immune mechanisms and chronic inflammatory cascades.	[46]
Four-Gene Model (*ADRB2* rs1042713, IL4 rs2243250, FCER1B rs569108, IL13 rs20541)	Asthma	Provides synergistic prediction of disease susceptibility and pediatric asthma risk; highlights pronounced ethnic heterogeneity.	[48,49]
Integrated Immunogenetic Profile (*ADRB2* + Immune Genes)	Asthma	Facilitates precise, multi-dimensional prediction of inter-individual variability in short-acting β-2 agonist (albuterol) efficacy.	[10,47]

## Data Availability

No new data were created or analyzed in this study. Data sharing is not applicable to this article.

## Abbreviations

The following abbreviations are used in this manuscript:

*ADRB2*	β-2 adrenergic receptor gene
SNPs	Single nucleotide polymorphisms
cAMP	Cyclic adenosine monophosphate
AS-PCR	Allele-specific polymerase chain reaction
β2AR	β-2 adrenergic receptor
GPCR	G protein-coupled receptor
Gs	G protein
F193	Phenylalanine 193
ECL2	Second extracellular loop
D113	Aspartate 113
N312	Asparagine 312
PKA	Protein kinase A
MLCK	Myosin light chain kinase
MAPK	Mitogen-activated protein kinase
ChIP	Chromatin immunoprecipitation
RT-qPCR	Quantitative real-time PCR
AC	Adenylyl cyclase
NK	Natural killer
PBMCs	Peripheral blood mononuclear cells
DCs	Dendritic cells
ILC2s	Group 2 innate lymphoid cells
Th2	T helper 2
Tregs	T cells
PCOS	Polycystic ovary syndrome
LABA	Long-acting β-2 agonist
ICS	Inhaled corticosteroids
COPD	Chronic obstructive pulmonary disease
